# Cost-effectiveness analysis of the human recombinant epidermal growth factor in the management of patients with diabetic foot ulcers

**DOI:** 10.1080/2000625X.2018.1480249

**Published:** 2018-06-26

**Authors:** Martin Romero Prada, Carolina Roa, Pamela Alfonso, German Acero, Lina Huérfano, David Vivas-Consuelo

**Affiliations:** a Public Health in Fundacion Salutia, Center in Health Economics Investigator, Bogotá D.C., Colombia; b Health Economics and management, Universitat Politècnica de València (UPV), Valencia, España

**Keywords:** Epidermal growth factor, diabetic foot, diabetes mellitus, wound healing, amputation

## Abstract

**Introduction**: Diabetic foot ulcers are one of the most frequent complications of diabetes; such ulcers cause an increase in the costs of the health care of the diabetic patient and can even cause disability due to amputation in the patient. Although a proportion of patients achieve a spontaneous closure of ulcers, others require medical or surgical treatment.

**Objective**: To determine the cost-effectiveness of the intra- and perilesional application of recombinant human epidermal growth factor (rhEGF), as opposed to conventional therapy for the management of patients diagnosed with Wagner’s 3 or 4 diabetic foot ulcer in Colombia.

**Methodology**: Using a Markov model, the process of care of a diabetic patient with diagnosis of Wagner’s 3 or 4 ulcer receiving conventional treatment, or intra- and perilesional rhEGF, is configured. The evaluation cycles of the treatments are weekly over a 5-year horizon and the outcomes evaluated are quality-adjusted life years (QALYs) and the number of amputations avoided by each treatment scheme, in addition to the total costs for treatments.

**Results**: For the analysed base case, in the outcome of amputations, it was found that the factor presents 39 fewer amputations, in a cohort of 100 patients, compared with conventional treatment. Likewise, QALYs are 0.65 more with the use of rhEGF in an average patient. The estimated cost-utility ratio for the base case would be below the threshold established for Colombia.

**Conclusions**: The intra- and perilesional application of rhEGF is a more effective therapeutic option than conventional therapy in the treatment of patients with Wagner’s 3 or 4 diabetic foot ulcers and is cost-effective, taking as an outcome the QALYs for Colombia from the perspective of the health system.

## Introduction

Diabetic foot is a clinical condition that can occur in people with diabetes mellitus, as a consequence of chronic endothelial damage, secondary to the chronic hyperglycaemic state [1]. Due to the neurological and vascular changes that diabetes triggers, these patients lose sensitivity in the extremities and have deformations in their feet, which increases the risk of having injuries that lead to ulcers, overinfections and, finally, amputations [1,2].

Regarding the origin of ulcers, 90% of these have diabetic neuropathy as a starting factor [3]. Within the neuropathy, there is the sensitive or motor type; the first is associated with traumatic injuries that do not generate pain, while the second can trigger malformations in the feet, which causes friction with the footwear and, in turn, leads to the formation of pressure ulcers. Additionally, these patients have fine or atrophic skin, which facilitates the formation of fissures and the entry of germs [2].

According to the Latin American Diabetes Association (LADA), ulcers and amputations in diabetic patients constitute an important problem for public health, since their costs are high, both for patients and their families and for the health system – in the latter case due to subsidies for disability conditions and costs for health care [2].

In the literature, prevalence for diabetic foot ulcers is reported from 4% to 10% in diabetic patients worldwide; of that percentage, between 60% and 80% of the cases are resolved, while between 5% and 24% end in the amputation of the limb [3]. In South and Central America, this indicator is between 5% and 20% [2], however; although the exact data of the diabetic foot epidemiology have not been documented, the high prevalence of the disease causes a greater risk of developing foot ulcers [4]. the prevalence of diabetic foot change according to age, gender and place of origin from 2.4% to 5.6% [5].

It is estimated that 24.4% of the total health expenditure of diabetic patients is due to complications related to the foot [4], and the cost of diabetic foot treatment is approximately $11 billion in the USA and $456 million in the UK [6]. Reported costs related to the management of diabetic foot ulcers that do not require amputations are between $993 and $17,519, compared to the cost of handling patients who required ulcer amputation, between $16,488 and $66,215 [4]; therefore, it is important to evaluate strategies that ensure better prevention and management of them.

The guidelines of the UK, on the management of diabetic foot, recommend taking measures to reduce pressure, control infections, control ischemia, debridement of wounds and perform wound care with gauze dressing as part of the treatment of the ulcer [7]. Adjuvant treatments for patients with diabetic foot ulcers include the use of growth factors, medicated dressings or negative pressure therapy, among others; electrical stimulation therapy and hyperbaric therapy with oxygen are also considered, although the latter still need clinical studies to be recommended in the treatment of ulcers [7].

The guide of the Colombian Diabetic Foot Group (COLPEDIS), within the treatment options of ulcers generated by this disease, includes the recombinant human epidermal growth factor (rhEGF), as an adjuvant in ulcer care to achieve better healing times [8]. The rhEGF is a peptide that promotes cell growth, proliferation, differentiation and survival that is related to the healing of wounds and maintenance of the integrity and regeneration of the skin.

In the case of diabetic foot ulcers generated by pressure and vascular origin, in which the balance of the growth factors in the tissues has been lost, therefore, the recombinant factor that stimulates the proliferation and migration of epithelial cells generates a closing of the chronic wound more quickly [9]. Given the above, its use in patients with diabetic foot ulcers has shown that it can prevent associated complications such as infection and amputations by up to 70% [10].

There is no evidence of its use for Colombia, nor studies to evaluate its long-term benefits, so this study seeks to analyse the cost-effectiveness of using the rhEGF in Colombian patients in such a way that it allows to contribute with objective data for the decision making in the country.

## Methodology

### Overview and treatment strategies

A cost-effectiveness analysis was developed to compare estimates of total costs of care and five-year clinical outcomes in a hypothetical cohort of diabetic patients diagnosed with Wagner’s 3 or 4 ulcer and controlled infection. This analysis evaluates two different strategies, the conventional treatment, comparing it with this same treatment but with the addition of the rhEGF. After a consultation with clinical experts, quality-adjusted life years (QALYs) were selected as an integral measure that groups the results in their different health and treatment states and, as an intermediate outcome, the amputations avoided, understood as the incremental among those estimated by the model when using rhEGF and those estimated with conventional therapy only.

The results are evaluated independently (costs and results) and as incremental cost-effectiveness ratio (ICER), for QALYs, because there is no threshold to evaluate the result for amputations avoided; this reason is obtained from quotient between the differences in effectiveness for each outcome and the difference between total costs.

A time horizon of 5 years was taken, a period that is considered to adequately evaluate the effects of treatment and evolution of the disease. No longer was taken because at the time of the study there are not the results of long-term studies for the technology.

The clinical data of the model were obtained after a scientific literature review of effectiveness and safety of the treatment [11,13]. Other comparators were not included because there are no studies comparing them head to head. The economic data were obtained from information on real transactions of the Colombian health system.

### Structure of the model

**Figure 1.**
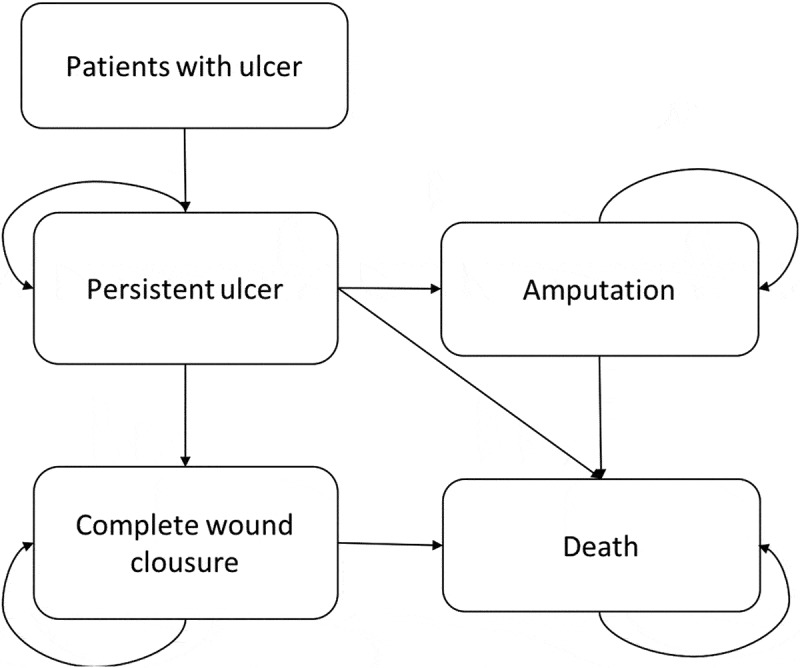
Markov model.

**Source**: design of the authors, 2017.

The model was developed in Microsoft Excel 2013 and reflects the natural history of the patient with diabetes Wagner’s 3 or 4 ulcer, with controlled infection, which is managed with conventional treatment. Patients travel between two states of health: the response status to the treatment of the ulcer, which can reach its complete closure, and the state of no response where the ulcer persists (Figure 1); according to the probabilities of transition that act in an exclusive manner, in weekly cycles since the factor is applied weekly during a period of 8 weeks, compared with conventional treatment that can extend up to 20 weeks [12,13]. Patients who are in the non-response state are affected by the probability of amputation and in turn these patients are affected by the probability of dying from amputation. In either of the two states, the patient may die from the disease.

### Clinical parameters

The clinical data related to the closure of the ulcer and amputation were calculated from the studies of Fernandez et al. [10,11], both for the conventional treatment strategy and for the growth factor. To transform the data of rates into proportions, the transformation formula of Fleurence and Hollenbeak [12] was used (see Table 1). The probabilities were adjusted according to the model cycles (1 week). The probability of amputation was taken from the Moulik study [13].

**Table 1.** Parameters included on the model.

Parameter	Base case values	Range sensibility analysis (20%)		Source
		Min.	Max.	
Week probability of complete wound closure with rhEGF 75 μg	0.0398	0.0318	0.0477	[12,13][10-13]
Week probability of complete wound closure with conventional therapy	0.0129	0.0103	0.0154	
Week probability of non-complete wound closure with rhEGF 75 μg*	0.9602	0.7682	1.0000	
Week probability of non-complete wound closure with conventional therapy*	0.9871	0.7897	1.0000	
Week probability of amputation with rhEGF 75 μg	0.0164	0.0131	0.0196	
Week probability of amputation with conventional therapy	0.0308	0.0246	0.0369	
Week probability of disease death (all the treatments evaluated)	0.0003	0.0002	0.0003	[14]
Week probability of death by amputation procedure (all the cases)	0.0029	0.0023	0.0035	[15]
Percentage of patients with prosthesis (over the knee)	100%	Colombian Health Maintenance Organization database		
Percentage of patients with prosthesis (under the knee)	15%	Colombian Health Maintenance Organization database		

*Author’s estimation (probability complementary to the probability of wound complete closure). rhEGF: recombinant human epidermal growth factor intra- or perilesional. Source: Chart developed from [1,2,4,5]

Based on the data reported in the literature, it is assumed within the model that, with the growth factor, the time at which we begin to see results of complete closure of the ulcer without recurrence is at 11 weeks, whereas with conventional therapy it is at 14 weeks [10].

### Economic parameters

This cost-effectiveness analysis was conducted from the perspective of the Colombian health system and the threshold of three times the gross domestic product (GDP), which is recommended by the Institute of Health Technology Evaluation of Colombia (IETS), was considered [14]. The GDP per capita in Colombia reported by the Bank of the Republic for 2016 was USD 17,696 for 2016. An average exchange rate for 2017 of Colombian pesos (COP) $2,951.35 = USD 1 [16] was considered. The costs and outcomes for the ICER estimates were adjusted with an annual discount rate of 5%, as suggested for Colombia [17].

### Costs

Costs associated with care for each clinical condition and associated controls were determined. This analysis only estimated the relevant direct costs, incurred in terms of the use of health resources, procedures and inputs, in each of the treatment options contemplated in the model.

The costs of amputation were determined in patients in whom the complete closure of the ulcer was not achieved; likewise, the cost of rehabilitation of the ulcer was included in the patients who responded to the treatment.

The costs of the prosthesis and rehabilitation for the amputees were calculated from information obtained from a health service provider in Colombia.

The costs of the technology were taken from the information reported in the Drug Price Information System (SISMED) for the first quarter of 2017, and the costs of health services from information on transactions within health insurance for 2016, which are the most recent at the moment of the development of the model, all reported in Colombian pesos (COP) and transformed into US dollars.

The cost of the epidermal growth factor includes the application costs that are estimated at around USD 144,876 (COP $434,628) per week (three applications).

The procedures and supplies used within the model were estimated based on the COLPEDIS clinical practice guideline [8], and the frequency of use for each treatment scheme was estimated based on the clinical experience of specialists in the area of interest (Table 2).

**Table 2.** Costs included within the model.

Item	Cost (USD)
rhEGF	
rhEGF + application (vial)	762,87
Total weeks rhEGF (3 vials weekly)	2.288,61
Weekly conventional therapy	
Wound care room	40,36
Wound care procedure	121,33
Sodium chloride solution	13,54
Gauze	135,39
Elastic bandages	26,91
Specialist counselling	5,51
Conventional therapy total per week	343,05
Others	
Prosthesis under the knee + procedure	1.965,22
Prosthesis over the knee	3.049,48
Amputation (average for toes, infracondylar or supracondylar amputation)	1.488,53
Rehabilitation for ulcer treated	408,80

Source: Chart developed by authors, 2017.

Regarding adverse events associated with the technologies under evaluation, no report or presentation evidence associated with the use of the technologies was found, so this analysis does not take them into account.

### Sensitivity analysis

A multivariate sensitivity analysis was developed to assess the robustness of the results and the uncertainty associated with the main parameters used to construct the model. The parameters of result were varied from −20% to +20% of the original value.

## Results

The results in an average patient with diabetic foot ulcer Wagner 3 or 4, who is treated with rhEGF compared to conventional therapy, showed that the QALYs obtained with the use of the factor are 3.98, while with conventional therapy they are 3.32. That is to say, that with the use of rhEGF, 0.65 QALYs are gained within the time horizon of 5 years (Table 3).

**Table 3.** Results of the base case rhEGF compared to conventional therapy.

Outcomes	Amputations	QALYs	Total Cost
Without discount			
rhEGF 75 μg	0.31	3.98	$19.024
Conventional therapy	0.7	3.32	$ 11.346
Incremental (per patient)			
rhEGF 75 μg	0.39	0.65	$7.678,61
Conventional therapy			
With discount			
rhEGF 75 μg	0.24	3.51	$18.953,73
Conventional therapy	0.56	2.93	$11.164,95
Discounted Incremental (per patient)			
rhEGF 75 μg			
Conventional therapy	0.32	0.58	$7.789

rhEGF: recombinant human epidermal growth factor intra- or perilesional. Source: Chart developed with the model results.

According to the model, per patient treated with rhEGF, there were 0.39 fewer amputations compared to conventional treatment (0.31 versus 0.70, respectively), also showing greater effectiveness.

From a cost point of view, the study showed that a patient treated with rhEGF would cost, on average, USD 19,024 against USD 11,346 when treated with conventional therapy. Table 4 shows the cost detail for the two arms, where it is possible to see the impact of the cost of the technology and the higher cost in the arm without the intervention of the amputations.

**Table 4.** Discriminated costs.

Concept	rhEGF USD	Conventional treatment USD	rhEGF	Conventional treatment
Amputee	1.145,03	2.684,19	6%	24%
Drugs	17.286,45	8.107,93	91%	71%
Procedures (non-closure wound patients)	592,77	553,52	3%	5%
TOTAL	19.024,25	11.345,64	100%	100%

Source: Chart developed with the model results.

**Figure 2.**
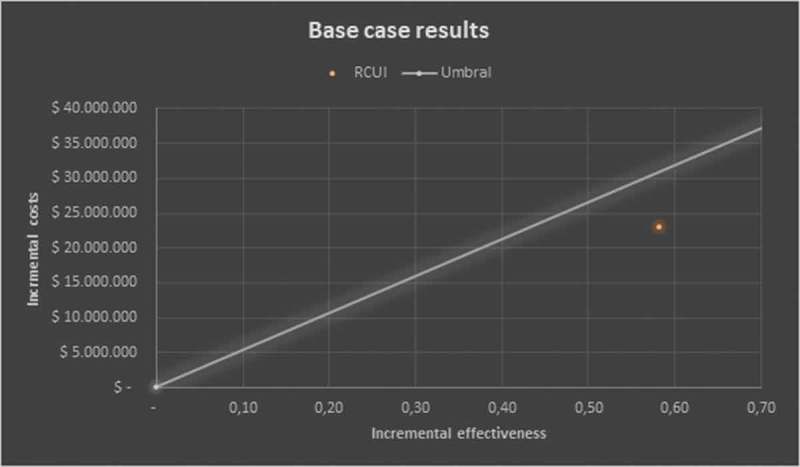
Results of the base case – cost-effectiveness plane.

Taking into account the incremental discounted, the estimated ICER would be USD 13,428.94 per QALYs, taking as a reference the threshold of 3 GDP per capita [16]; the use of the factor would be located in the cost-effectiveness quadrant below the threshold which shows it as cost-effective (Figure 2).

**Figure 3.**
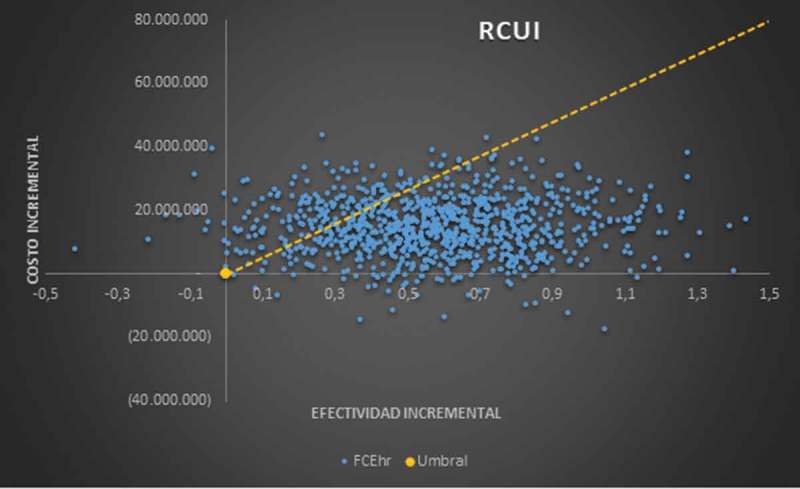
Monte Carlo analysis.

The multivariate sensitivity analysis run in 1,000 iterations, modifying all the variables included in the model, show that cost-effectiveness remains at 78.1% (below the threshold) and only 16.4% would be located above the threshold, additionally 4.2% are dominant (Figure 3).

## Discussion

This study demonstrates the cost-effectiveness and cost utility of rhEGF compared to conventional treatment in the Colombian context, where there are no studies of this type for patients with diabetic foot ulcers.

the growth factors have evidence that shows that they achieve better results in the closure of ulcers than not using them, as the study by Martí-Carvajal et al. shows [18] is, one of the advantages of rhEGF compared to conventional therapy is the treatment time, since with the application for 8 weeks the complete closure of the ulcer is obtained on average at 14 weeks [10,12,13] in 75.5% of the cases, while in conventional therapy the complete closure of the ulcer is achieved only in 52.1% of the cases after an average treatment of 20 weeks [10,12,13].

During the approach of the present study, the analysis was contemplated against the medicated dressings and negative pressure therapy which, according to the clinical experts consulted and the different clinical practice guidelines analysed, are not comparable with the technology in evaluation given which are used at different times during the treatment of this clinical condition. That is, the target population for the use of treatments has different characteristics, so they were not included in the present economic evaluation [2,7,8].

Within the limitations of the study, the main one could be that the utilities used within the evaluation carried out are not estimated for the Colombian population, although it is accepted as valid the use of the same made in other countries as long as they are made according to measures based on preferences, as is the case of those used in this study. Likewise, it would have been important to analyse against other therapies or make a wider gap in the differences between conventional therapies, but the information available from evidence of clinical studies found does not allow it and would only be possible with real life studies not available at the time. Additionally, the inclusion of standard treatment as a comparator within the economic evaluation is based on the opinion of Colombian clinical experts, who suggested the management of patients according to their criteria in clinical practice.

This cost-effectiveness analysis demonstrated in the base case (using a discount rate of 5% per year) that the use of rhEGF is a cost-effective option in the two evaluated outcomes, with 0.39 amputations avoided with the use of the technology and 0.65 QALYs gained versus conventional therapy, showing results similar to those presented in the study by Acosta et al. [19], where it was demonstrated that the use of rhEGF reduces amputations by 11.8% when compared to standard therapy and obtains 1.58 months of life adjusted for quality over the 1-year horizon. In this time horizon, the results are lower than those obtained within this 5-year economic evaluation, which reveals that in the long term the benefits of the evaluated technology obtain better results for the patient in this outcome.

The present economic evaluation was developed from the perspective of the Colombian health system in terms of cost-effectiveness, which suggests that only the direct costs assumed by the third-party payer should be taken into account; therefore, the costs that are not related were not taken into account directly to the use of technologies, nor the out-of-pocket costs (which are contemplated in the cost-benefit assessment), nor the costs associated with the recurrences of the disease with their respective hospital readmissions, unlike the analysis conducted by Acosta et al. [19], where the indirect costs of temporary or permanent disability of the amputations are analysed. That said, it is important to recognize that more than 80% of the costs in the case of rhEGF would be subject to recovery by health insurers, so in an analysis from the insurer’s perspective, its use could be seen as dominant compared to conventional therapy for being cost-saving and more effective.

In the sensitivity analysis made for the outcome of QALYs, it is evident that the cost-effectiveness of the rhEGF is maintained in 78.1% of cases, and that for 16.4%, the factor is no longer cost-effective, being located above the threshold, also demonstrating that the rhEGF can be dominant compared to the conventional option in 4.2% of the scenarios contemplated.

One of the components to be highlighted is the price of the prosthesis used in the evaluation which, although it is an average price according to the data found within the available bases of providers in the country, is very low compared to the one prostheses have at the international level, as shown by the study by Rodriguez et al. [20], where the average cost of a prosthesis for patients of this type is USD 25,000 for 2007, which is equivalent to approximately 52 million COP. Currently, due to the appreciation of the dollar against the COP, this value is much higher.

In future analyses, it would be worthwhile to include 5-year mortality after amputation, which can be between 50% and 60% [4,21]; these data show that amputations are a fundamental factor in evaluating the treatment that a patient with diabetic foot ulcer should receive. It is also important to include a parameter of hospital recurrences and readmissions that have not been taken into account in this evaluation due to the lack of evidence to corroborate the reality of clinical practice [22].

## Conclusions

The results of this study show that rhEGF would avoid amputations in patients with diabetic foot ulcers with approximately 32 fewer amputations, in a cohort of 100 patients, compared to treatment with conventional therapy and an increase in quality of life represented in a gain of 0.58 years adjusted for quality with an incremental of COP $22,987,212.

As a consequence of what has been previously analysed, rhEGF is a cost-useful alternative in the care of patients with Wagner 3 or 4 diabetic foot ulcers compared to conventional treatment received by these patients. Likewise, the results remain unchanged when carrying out the sensitivity analysis.

## References

[1] Nehring P, Mrozikiewicz-Rakowska B, Krzyżewska M, et al Diabetic foot risk factors in type 2 diabetes patients: a cross-sectional case control study. J Diabetes Metab Disord [Internet]. 2014;13:79. Available from: http://www.pubmedcentral.nih.gov/articlerender.fcgi?artid=4128535&tool=pmcentrez&rendertype=abstract 10.1186/2251-6581-13-79 PMC4128535 25114882

[2] Mesa Perez JA, Vitarella G, Rosas Guzman J Guías ALAD de Pie Diabético. Rev ALAD. 2010;XVIII(2):73–85.

[3] Alexiadou K, Doupis J. Management of diabetic foot ulcers. Diabetes Ther [Internet]. 2012;3(1):4. Available from: http://www.ncbi.nlm.nih.gov/pubmed/22529027 10.1007/s13300-012-0004-9 PMC3508111 22529027

[4] Boulton AJM, Vileikyte L, Ragnarson-Tennvall G, et al. The global burden of diabetic foot disease. Lancet. 2005;366(9498):1719–1724. 10.1016/S0140-6736(05)67698-2 16291066

[5] Tirado RA del C, López JAF, Tirado FJ del C. Guía de práctica clínica en el pie diabético. Arch Med. 2014;10(1):1–17.

[6] Al-Rubeaan K, Al Derwish M, Ouizi S, et al. Diabetic foot complications and their risk factors from a large retrospective cohort study. PLoS One. 2015;10(5):1–17. 10.1371/journal.pone.0124446 PMC4422657 25946144

[7] NICE. Diabetic foot problems : prevention and management. NICE Guidel [Internet]. 2015;(August). Available from: http://www.nice.org.uk/guidance/ng19/resources/diabetic-foot-problems-prevention-and-management-1837279828933

[8] Júbiz PY, Márquez SG, Márquez ZA,. Guías colombianas para la prevención, diagnóstico y tratamiento del pie diabético 2012. COLPEDIS, Grup Colomb Pie Diabético. 2012;1:98. . p. 1–7.

[9] Esquirol J, Vila H. Factor de crecimiento epidérmico, innovación y seguridad Factor de crecimiento epide. Med Clin. 2014;145(7). 10.1016/j.medcli.2014.09.012 25433777

[10] Gomez-Villa R, Aguilar-Rebolledo F, Lozano-Platonoff A, et al. Efficacy of intralesional recombinant human epidermal growth factor in diabetic foot ulcers in Mexican patients: A randomized double-blinded controlled trial. Wound Repair Regen. 2014;22(4):497–503. 10.1111/wrr.12187 25041620

[11] Redekop WK, Stolk EA, Kok E, et al Diabetic foot ulcers and amputations: estimates of health utility for use in cost-effectiveness analyses of new treatments. Diabetes Metab. 2004;30:549–556. 15671925 10.1016/s1262-3636(07)70154-4

[12] Fernández-Montequín JI, Valenzuela-Silva CM, Díaz OG, et al. Intra-lesional injections of recombinant human epidermal growth factor promote granulation and healing in advanced diabetic foot ulcers: Multicenter, randomised, placebo-controlled, double-blind study. Int Wound J. 2009;6(6):432–443. 10.1111/j.1742-481X.2009.00641.x PMC7951641 20051095

[13] Fernández-Montequín JI, Infante-Cristiá E, Valenzuela-Silva C, et al. Intralesional injections of Citoprot-P® (recombinant human epidermal growth factor) in advanced diabetic foot ulcers with risk of amputation. Int Wound J. 2007;4(4):333–343. 10.1111/j.1742-481X.2007.00344.x PMC7951380 17953679

[14] Malyar N, Freisinger E, Meyborg M, et al. Amputations and mortality in in-hospital treated patients with peripheral artery disease and diabetic foot syndrome. J Diabetes Complications [Internet]. 2016; Available from: http://www.sciencedirect.com/science/article/pii/S1056872716300897 10.1016/j.jdiacomp.2016.03.033 27118161

[15] Moulik PK, Mtonga R, Gill GV. Amputation and mortality in new-onset diabetic foot ulcers stratified by etiology. Diabetes Care. 2003;26(2):491–494. 10.2337/diacare.26.2.491 12547887

[16] Banco de la República. Tasa de cambio del peso colombiano (TRM) [Internet]. http://www.banrep.gov.co. 2016 p. 4. Available from: http://www.banrep.gov.co/es/trm

[17] Moreno Viscaya M, Mejía Mejía A, Castro Jaramillo HE. Manual para la elaboración de evaluaciones económicas en salud. Instituto de Evaluación Tecnológia en Salud. 2014.

[18] Martí-Carvajal AJ, Gluud C, Nicola S, et al. Growth factors for treating diabetic foot ulcers. Vol. 2015, Cochrane Database of Systematic Reviews. 2015. 10.1002/14651858.CD008548.pub2 PMC8665376 26509249

[19] Acosta-reyes MR, López A, Álvarez O, González AM, et al. Evaluación económica del uso de factor de crecimiento epidérmico recombinante humano (FCErh) en población mexicana con pie diabético en el Sector Salud. 2014;81(3):147–53.

[20] Rodriguez D, Erika A. Estudio y análisis comparativo de mercados de prótesis médicas de fabricación colombiana para la exportación a China o Estados Unidos. Univ San Buenaventura [Internet]. 2007; Available from: http://biblioteca.usbbog.edu.co:8080/Biblioteca/BDigital/40980.pdf

[21] López-saura PA. Intralesional Human Recombinant Epidermal Growth Factor for the Treatment of Advanced Diabetic Foot Ulcer : From Proof of Concept to Confirmation of the Efficacy and Safety of the Procedure. 2006.

[22] Reiber, G. E. “The epidemiology of diabetic foot problems.” Diabetic medicine: a journal of the British Diabetic Association13 (1996): p S6. 8741821

